# The Impact of Chronic Kidney Disease and Short-Term Treatment with Rosiglitazone on Plasma Cell-Free DNA Levels

**DOI:** 10.1155/2014/643189

**Published:** 2014-10-13

**Authors:** Amanda L. McGuire, Nadia Urosevic, Doris T. Chan, Gursharan Dogra, Timothy J. J. Inglis, Aron Chakera

**Affiliations:** ^1^School of Medicine and Pharmacology, University of Western Australia, Nedlands, WA, Australia; ^2^Harry Perkins Institute of Medical Research, QEII Medical Centre, Nedlands, WA, Australia; ^3^Department of Microbiology, PathWest Laboratory Medicine WA, Nedlands, WA, Australia; ^4^Department of Renal Medicine, Sir Charles Gairdner Hospital, Nedlands, WA, Australia; ^5^School of Pathology and Laboratory Medicine, University of Western Australia, Nedlands, WA, Australia; ^6^Translational Renal Research Group, Harry Perkins Institute of Medical Research, 5th Floor QQ Block, QEII Medical Centre, 6 Verdun Street, Nedlands 6009, Australia

## Abstract

Patients with chronic kidney disease (CKD) are at increased risk of cardiovascular disease. Circulating free nucleic acids, known as cell-free DNA (cfDNA), have been proposed as a novel biomarker of cardiovascular risk. The impact of renal impairment on cfDNA levels and whether cfDNA is associated with endothelial dysfunction and inflammation in CKD has not been systematically studied. We analysed cfDNA concentrations from patients with varying degrees of CKD. In addition, to determine whether there is a relationship between cfDNA, inflammation, and endothelial dysfunction in CKD, levels of proinflammatory cytokines and von Willebrand Factor (vWF) were measured in patients treated with the peroxisome proliferator-activated receptor gamma agonist rosiglitazone or placebo for 8 weeks. cfDNA levels were not increased with renal impairment or associated with the degree of renal dysfunction (*P* = 0.5). Treatment with rosiglitazone for 8 weeks, but not placebo, was more likely to lead to a reduction in cfDNA levels (*P* = 0.046); however, the absolute changes in cfDNA concentrations during treatment were not statistically significant (*P* > 0.05). cfDNA levels correlated with markers of endothelial dysfunction (hsCRP *P* = 0.0497) and vWF (*P* = 0.0005). In conclusion, cell-free DNA levels are not influenced by renal impairment but do reflect endothelial dysfunction in patients with CKD.

## 1. Introduction

Patients with renal impairment have an elevated risk of cardiovascular disease, which increases as renal function declines [1, 2]. Recently, the presence of free nucleic acids in the peripheral circulation, referred to as cell-free DNA (cfDNA), has been proposed as a novel biomarker of cardiovascular risk [3], with elevated levels also reported in a variety of inflammatory states, including treatment with haemodialysis [4–11]. The development of noninvasive measures to quantify this risk and therapies that may reduce this risk are therefore of significant clinical interest. Several small case series have suggested that cfDNA levels are not affected by renal dysfunction; however, the effect of different degrees of renal impairment has not been systematically studied nor has the impact of anti-inflammatory agents on cfDNA levels. As cfDNA levels may provide a way to noninvasively monitor disease activity and to predict clinical outcomes [10], determining whether cfDNA levels reflect the elevated cardiovascular risk and endothelial dysfunction that occurs with CKD is of clinical relevance.

As chronic kidney disease (CKD) is considered a proinflammatory state [12, 13], we hypothesised that cfDNA levels would be elevated in patients with renal impairment and that treatments that decreased endothelial dysfunction would reduce cfDNA levels. To answer these questions, we measured plasma cfDNA levels in 127 patients with a wide range of renal impairment (CKD stages 2–5) who participated in two randomized controlled trials that investigated vascular function and inflammation in patients with CKD [14, 15]. In addition to quantifying the total amount of cfDNA, as apoptotic cfDNA may have specific effects on endothelial cells, in a subset of patients the proportion of apoptotic cfDNA (fragments sizes of 160–200 bp) was assessed [16]. Next, to determine if there was a link between cfDNA levels and endothelial dysfunction and inflammation in CKD [17–20], we measured concentrations of cfDNA and inflammatory cytokines in 70 patients treated with either placebo or the peroxisome proliferator-activated receptor gamma (PPAR*γ*) agonist rosiglitazone for 8 weeks [15].

## 2. Materials and Methods

### 2.1. Patient Cohorts

Clinical samples were obtained from participants in two trials referred to internally as REVERT [15] and SAFIRE [14]. REVERT was a randomized, double-blind placebo-controlled study comparing 8-week treatment with rosiglitazone 4 mg daily in 70 patients with stages 3-4 CKD. Plasma samples were available for all 70 subjects at baseline and week 8 (140 samples in total). SAFIRE was a randomized, double-blind placebo-controlled parallel group study comparing atorvastatin, 40 mg daily (*n* = 31), gemfibrozil, 600 mg twice daily (*n* = 27), and placebo (*n* = 32) for 6 weeks in patients with stages 3–5 CKD. (Following screening, several patients in both studies had baseline eGFRs between 60 and 90 mL/min/1.73 m^2^ and were therefore classified as CKD stage 2.) Plasma samples were available for 19 patients in the atorvastatin group, 17 from the gemfibrozil group, and 21 who received placebo at baseline and week 6 (114 samples in total). 26% of the SAFIRE subjects were on haemodialysis and 14% on peritoneal dialysis. This study was approved by the Ethics Committees of Royal Perth and Sir Charles Gairdner Hospitals. Written informed consent was obtained from all participants and research was conducted in accordance with the Declaration of Helsinki.

### 2.2. Plasma cfDNA Isolation

Cell-free DNA was isolated using a QIAamp DNA Blood Mini Kit (Qiagen) with modifications as described previously [21]. Plasma samples were obtained from either citrate or EDTA collections, which with our protocol yield equivalent cfDNA levels (see Supplementary Figure 1 in Supplementary Material available online at http://dx.doi.org/10.1155/2014/643189). Briefly, plasma samples were thawed at room temperature (RT), inverted to mix, and centrifuged at maximum speed for 10 minutes at 4°C in a microcentrifuge. 200 *μ*L aliquots of plasma were removed for cfDNA isolation and incubated with RNase A and Proteinase K before binding of cfDNA to the supplied columns. Two wash steps were performed; then cfDNA was eluted with 50 *μ*L of nuclease-free water (incubated for 5 minutes at room temperature before centrifugation), and the eluate passed through the membrane a second time to maximise recovery. cfDNA was stored at 4°C for up to a month or at −20°C for extended storage.

### 2.3. cfDNA Quantification

cfDNA was quantified using the Qubit dsDNA HS Assay Kit (Life Technologies, CA) according to the manufacturer's protocol. cfDNA samples (10 *μ*L) were added to Qubit Working Solution (190 *μ*L), vortexed for 5 seconds, and incubated at RT for 2 minutes. A Qubit 2.0 Fluorometer (Life Technologies, CA) was used to calculate the dsDNA concentration.

### 2.4. cfDNA Analysis

To determine the amount of apoptotic cfDNA present, 1 *μ*L aliquots of cfDNA were analysed on Agilent DNA 12,000 chips using an Agilent 2100 Bioanalyzer (Agilent Technologies, California, USA) according to the manufacturer's instructions. All sample wells contained two internal markers of known size (50 bp and 17 kb) and DNA ladder.

### 2.5. Cytokine Measurements

The cytokines IL-1*β*, IL-6, IL-8, IL-10, and TNF*α* were quantified using the Milliplex MAP High Sensitivity Human Cytokine magnetic bead panel (EMD Millipore Corp., St. Charles, MO, USA) with plates read on a Luminex 200 system using xPonent software, version 3.1 (Luminex Corporation, Austin, TX, USA). 50 *μ*L of plasma was analysed per well, and all samples were assayed in duplicate as per the manufacturer's recommendation. Normalisation of data from different assay plates was performed by Merck Millipore (Frenchs Forest, NSW, Australia). Data for IL-10 was subsequently excluded from analyses due to excessive variance from the reported sensitivity and performance of the IL-10 assay. To confirm the accuracy of the Luminex system, IL-6 results were compared with those obtained using a quantitative enzyme immunoassay (Quantikine HS; R&D Systems Inc., Minneapolis, MN) and found to be highly correlated (*P* < 0.0001). von Willebrand factor (vWF) activity was measured with the ATA-Liatest vWF kit (Diagnostica Stago, Parsippany, NJ) and Diagnostica Stago STAR-automated coagulation analyser. High sensitivity C-reactive protein (hsCRP) levels were determined with an immunonephelometric method (Dade Behring Marburg GmbH, Marburg, Germany).

### 2.6. Statistical Analysis

Statistical analyses were performed using Prism 6 (Graphpad Software, La Jolla, CA, USA). Normality was assessed using the D'Agostino and Pearson omnibus test. Corrections for multiple comparisons were performed using Dunn's test. Statistical significance was defined as *P* < 0.05.

## 3. Results

**Figure 1:**
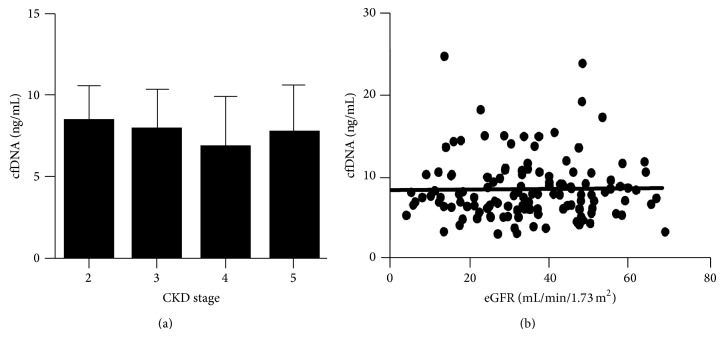
Cell-free DNA levels in patients with renal impairment. (a) Comparison of cfDNA levels by CKD stage (mean and SD are shown). No difference in any group was evident (*P* = 0.49). (b) Correlation between estimated glomerular filtration rate (calculated using the 4-variable MDRD equation) and cfDNA levels for individual patients is shown (*r* ^2^ = 0.0002, *P* = 0.87).

Baseline cfDNA levels from REVERT and SAFIRE were grouped by CKD status (stages 2–5) and the mean, median, and interquartile ranges calculated. One hundred and twenty-seven samples had baseline cfDNA results available; one sample (from a patient with CKD stage 5) was excluded from further analyses, as cfDNA levels were more than 10-fold higher than other values. Median cfDNA levels for each CKD stage were 2 (8.4 ng/mL), 3 (7.9 ng/mL), 4 (6.8 ng/mL), and 5 (7.7 ng/mL) (Figure 1(a)). There were no significant differences in cfDNA levels between any of the CKD stages (*P* = 0.5). As separating patients by CKD stage could reduce the ability to detect a more subtle relationship between cfDNA and renal function, the association between cfDNA levels and individual patient's estimated glomerular filtration rates (eGFR) was explored. No correlation between cfDNA and eGFR levels was evident (*P* = 0.87) (Figure 1(b)).

cfDNA can be derived from necrotic or apoptotic cells, which may reflect the underlying processes leading to its production and influence its biological properties [16]. To determine the relative proportion of apoptotic versus necrotic cfDNA in our patients, a subset of 12 plasma samples from both the SAFIRE and REVERT cohorts (including patients whose cfDNA levels remained constant, increased, or decreased at follow-up) were analysed on Agilent DNA 12,000 chips. No apoptotic DNA fragments were present in any of the samples tested (Supplementary Figure 2).

**Figure 2:**
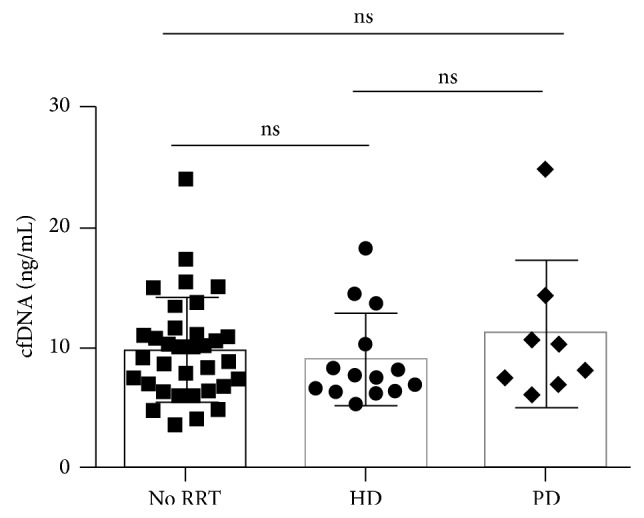
Influence of renal replacement therapy on cfDNA levels. Comparison of cfDNA levels from patients not on renal replacement therapy (RRT) with patients on haemodialysis (HD) or peritoneal dialysis (PD) who participated in the SAFIRE trial. Mean + SD and absolute values are shown (*P* = 0.6).

Haemodialysis is an intermittent therapy, which can lead to the release of inflammatory mediators, and has been shown to cause an increase in cfDNA levels [11]. Whether similar changes occur in patients receiving peritoneal dialysis (a continuous therapy) is unclear. To answer this question we measured cfDNA levels in 57 patients from the SAFIRE cohort. All samples from haemodialysis patients were collected prior to treatment. There was no difference in baseline cfDNA levels between subjects undergoing haemodialysis (average: 9.0 ng/mL, median: 7.6 ng/mL) or peritoneal dialysis (average: 11.1 ng/mL, median: 9.2 ng/mL) or those individuals not on dialysis (average: 9.8 ng/mL, median: 9.6 ng/mL) (*P* = 0.6) (Figure 2).

**Figure 3:**
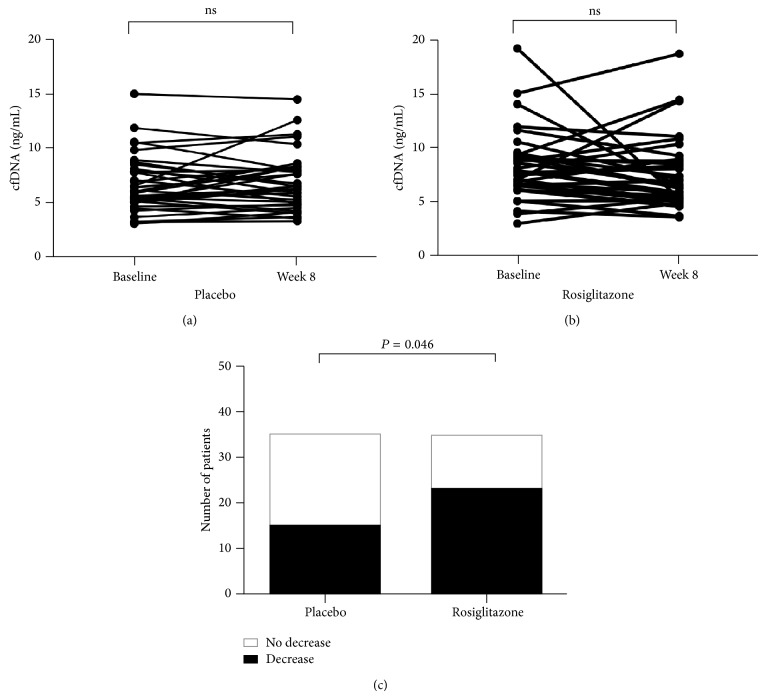
Changes in cfDNA levels. cfDNA levels were measured at baseline and following 8-week treatment with either placebo (a) or rosiglitazone (b). There were no differences between levels at baseline or follow-up in either group (*P* = 0.79 for placebo and *P* = 0.11 for rosiglitazone). However, the proportion of patients who had a decrease in their cfDNA levels over the course of the trial was significantly higher in the rosiglitazone-treated group (*P* = 0.046), shown in (c) as the filled area.

The PPAR*γ* agonist rosiglitazone has anti-inflammatory properties, which might influence the generation of cfDNA providing a link between cfDNA, inflammation, and vascular dysfunction in CKD. Therefore, we next compared cfDNA levels at baseline and following 8-week treatment with either placebo or rosiglitazone. No difference in cfDNA levels from baseline was detected in either group (*P* = 0.7864 and *P* = 0.1082 for placebo and rosiglitazone, resp.; see Figure 3). However, on follow-up patients randomized to rosiglitazone were significantly more likely to show a decrease in cfDNA levels (Figure 3(c); *P* = 0.046).

**Figure 4:**
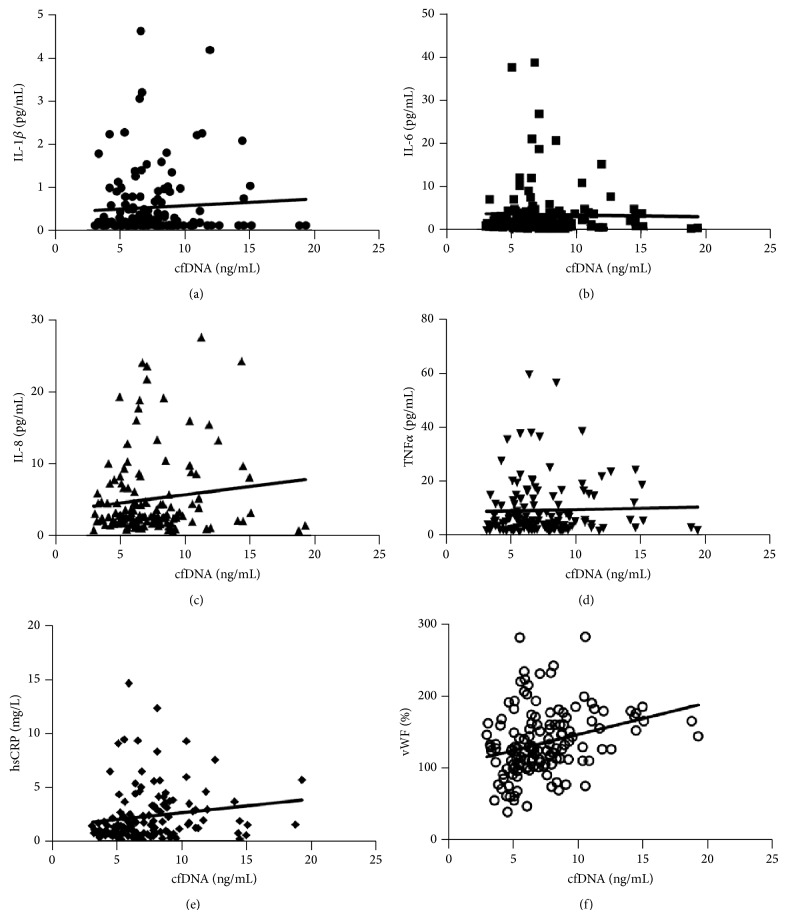
Correlation between cfDNA and cytokine levels. (a) IL-1*β*, *P* = 0.48; (b) IL-6, *P* = 0.81; (c) IL-8, *P* = 0.15; (d) TNF*α*, *P* = 0.75; (e) hsCRP, *P* = 0.0497; and (f) vWF, *P* = 0.0005.

To further assess whether there is a relationship between cfDNA levels, inflammation, and CKD, concentrations of cytokines linked to inflammation and endothelial dysfunction (IL-1*β*, IL-6, IL-8, TNF*α*, hsCRP, and vWF) were compared with levels of cfDNA in all patients from the REVERT cohort. For IL-1*β*, 56 of the 140 samples (40%) had at least one data point of ≤0.14 pg/mL, which was the limit of detection for this assay. For IL-6, 7 of the 140 samples (5%) had at least one data point of ≤0.14 pg/mL, which was the limit of detection for this assay. All data points for IL-8, TNF*α*, hsCRP, and vWF had detectable levels of the respective cytokines. Results stratified by CKD stage are shown in Table 1. No relationship between cfDNA and levels of IL-1*β*, IL-6, IL-8, or TNF*α* was identified (*P* > 0.1 for all cytokines) (Figures 4(a)–4(d)). However, cfDNA levels were correlated to levels of hsCRP (*P* = 0.0497) and vWF (*P* = 0.0005), which have previously been shown to reflect endothelial dysfunction and associate with elevated cardiovascular risk (Figures 4(e) and 4(f)) [15, 22, 23].

## 4. Discussion

We report the largest study to date analysing the effect of renal impairment on circulating cfDNA levels and demonstrate that, even in the presence of advanced kidney disease, levels of cfDNA are equivalent to those reported in healthy controls and that apoptotic cfDNA fragments are not present. Eight-week treatment with the peroxisome proliferator-activated receptor (PPAR) agonist rosiglitazone, an agent which has been demonstrated to reduce inflammation and endothelial dysfunction in patients with CKD [15], was associated with an increased likelihood of a reduction in cfDNA levels compared to placebo, but the absolute changes were not statistically significant. cfDNA levels correlated with markers of endothelial dysfunction (hsCRP *P* = 0.0497) and vWF (*P* = 0.0005) but not with proinflammatory cytokines in patients with CKD.

Previous studies in patients with end stage kidney disease have shown that cfDNA levels are increased within a few minutes of commencing haemodialysis but that baseline levels are generally equivalent to those found in healthy controls [11, 24]. However, a wide variation in normal ranges has been reported, and comparison between studies is problematic as results of cfDNA assays are highly dependent upon sample collection and processing methods [25, 26]. Using a validated protocol for cfDNA isolation and quantification [21], we analysed cfDNA levels in patients with moderate to severe renal impairment, including patients requiring dialysis. Consistent with previous data, cfDNA levels in patients with CKD stages 2–5, including dialysis dependent patients, were equivalent to those reported for healthy controls [27–30] and no association between eGFR and cfDNA concentrations was detected (Figure 1(b)). In addition, apoptotic DNA fragments were not present, suggesting that the elevated cardiovascular risk associated with CKD is not driven by processes that drive apoptosis or that the amount of apoptosis is insufficient to overwhelm clearance mechanisms [24, 31].

Endothelial dysfunction is an early finding in atherosclerosis and is strongly associated with inflammation and insulin resistance [32, 33]. PPAR agonists are insulin-sensitizing agents marketed for the treatment of type 2 diabetes mellitus that can improve endothelial function and inhibit the development of atherosclerosis [34–36]. Although enthusiasm for their use diminished following publication of a meta-analysis suggesting an increased risk of myocardial infarction in patients treated with rosiglitazone [37], subsequent studies have not supported this finding [38, 39], leading to a resurgence in interest in these agents. In the REVERT trial, short-term rosiglitazone therapy significantly lowered insulin resistance, high sensitivity C-reactive protein (hsCRP), and von Willebrand factor (vWF) [15], suggesting that PPAR*γ* agonists may reduce endothelial dysfunction in patients with CKD. In keeping with this hypothesis, we demonstrated that patients who received rosiglitazone were more likely to show a decrease in cfDNA levels after 8 weeks than those who received placebo (although absolute differences in cfDNA levels were not statistically significant) and that cfDNA levels correlated to levels of vWF and cfDNA, established markers of endothelial dysfunction [40, 41].

There are several limitations to our study. Despite the use of a high sensitivity cytokine detection kit and an optimized protocol for the detection of cfDNA, levels of cfDNA and several cytokines were at the lower limits of detection, which may have reduced the ability to detect small differences compared to background. Eight-week therapy with rosiglitazone may have been insufficient for meaningful differences in cfDNA levels to develop or the anti-inflammatory benefits of this agent may only become evident in the presence of more overt inflammation where levels of cytokines and cfDNA are orders of magnitude higher [10, 42]. Further prospective studies will be required to answer these questions and to resolve whether PPAR agonists have a therapeutic role in inflammatory states [43–46].

## 5. Conclusions

Chronic kidney disease is an inflammatory state associated with increased cardiovascular morbidity and mortality. cfDNA levels are elevated in the presence of inflammation, have been proposed as a biomarker for cardiovascular risk and a tool to monitor inflammatory conditions, and have intrinsic proinflammatory properties. The impact of renal impairment on cell-free DNA levels has not previously been systematically studied. The stage of CKD does not influence levels of cfDNA; however, there is a correlation with levels of hsCRP and vWF, but not other inflammatory cytokines associated with endothelial dysfunction. PPAR*γ* agonists may have a role in reducing cfDNA levels. cfDNA levels do not reflect the elevated cardiovascular risk in patients with CKD but may therefore retain their utility as a biomarker for other inflammatory conditions in patients with renal impairment.

## Supplementary Material

Supplementary Figure 1: Plasma cell cfDNA levels are equivalent in EDTA or citrate-containing tubes. cfDNA levels were calculated in triplicate samples processed independently from two healthy controls. No differences were detected between samples collected in EDTA or citrate-containing tubes.
Supplementary Figure 2: Apoptotic cfDNA is not present in patients with CKD. 12 cfDNA samples from patients in the REVERT and SAFIRE studies were run on Agilent DNA 12,000 chip to determine the proportion of apoptotic cfDNA present. Panel A) shows a microfluidic DNA gel with a representative electropherogram from one of these samples in Panel B). Panels C) and D) show equivalent traces from patients with sepsis, arrows indicate apoptotic DNA peaks.

## Figures and Tables

**Table 1:** Baseline characteristics of the study population by CKD stage.

	CKD stage			
	2	3	4	5
	Median (SD)	Median (SD)	Median (SD)	Median (SD)
cfDNA (ng/mL)	8.4 (2.80)	7.9 (3.87)	6.8 (3.05)	7.7 (4.86)
hsCRP (mg/L)	1.28 (2.38)	1.79 (2.25)	1.58 (1.85)	1.14 (1.66)
vWF (%)	126 (26.5)	135 (35.6)	138.5 (27.9)	129 (22.4)
IL-1*β* (pg/mL)	0.21 (2.3)	0.15 (0.78)	0.16 (0.79)	0.17 (0.55)
IL-6 (pg/mL)	1.57 (3.8)	1.3 (5.2)	1.7 (2.2)	1.52 (3.7)
IL-8 (pg/mL)	2.16 (3.7)	2.66 (5.1)	2.94 (4.3)	2.91 (6.01)
TNF*α* (pg/mL)	2.30 (11.3)	5.40 (8.5)	4.66 (9.64)	5.40 (11.7)
