# Supplementary figures and images for: The Impact of Chronic Kidney Disease and Short-Term Treatment with Rosiglitazone on Plasma Cell-Free DNA Levels
Source: PPAR Res. 2014 Oct 13;2014:643189. doi: 10.1155/2014/643189 (PMC4211161; doi:10.1155/2014/643189)

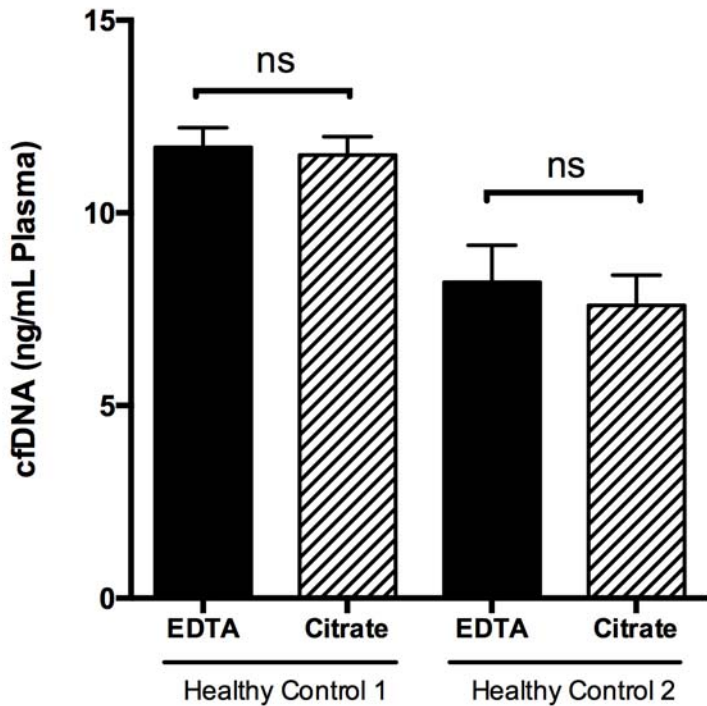

Supplement: Supplementary file 1 — Supplementary Figure 1: Plasma cell cfDNA levels are equivalent in EDTA or citrate-containing tubes. cfDNA levels were calculated in triplicate samples processed independently from two healthy controls. No differences were detected between samples collected in EDTA or citrate-containing tubes. Supplementary Figure 2: Apoptotic cfDNA is not present in patients with CKD. 12 cfDNA samples from patients in the REVERT and SAFIRE studies were run on Agilent DNA 12,000 chip to determine the proportion of apoptotic cfDNA present. Panel A) shows a microfluidic DNA gel with a representative electropherogram from one of these samples in Panel B). Panels C) and D) show equivalent traces from patients with sepsis, arrows indicate apoptotic DNA peaks. [file 643189.f1.zip › Supplementary_Figure_1.pdf]

**A**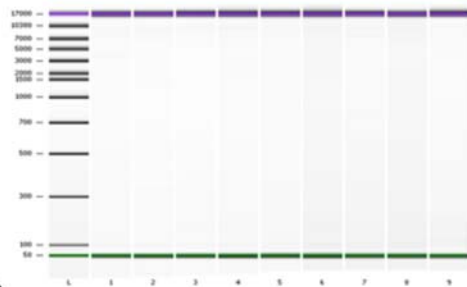**B**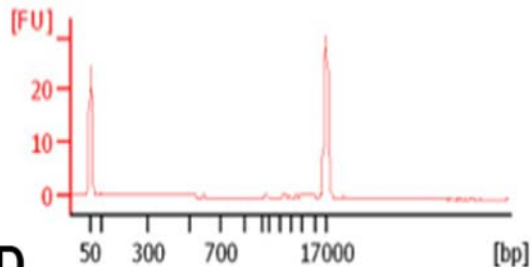**C**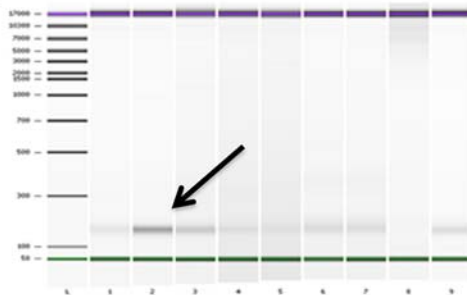**D**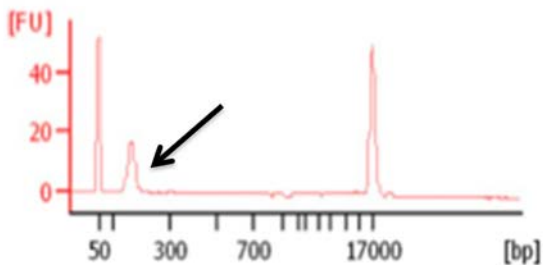

Supplement: Supplementary file 1 — Supplementary Figure 1: Plasma cell cfDNA levels are equivalent in EDTA or citrate-containing tubes. cfDNA levels were calculated in triplicate samples processed independently from two healthy controls. No differences were detected between samples collected in EDTA or citrate-containing tubes. Supplementary Figure 2: Apoptotic cfDNA is not present in patients with CKD. 12 cfDNA samples from patients in the REVERT and SAFIRE studies were run on Agilent DNA 12,000 chip to determine the proportion of apoptotic cfDNA present. Panel A) shows a microfluidic DNA gel with a representative electropherogram from one of these samples in Panel B). Panels C) and D) show equivalent traces from patients with sepsis, arrows indicate apoptotic DNA peaks. [file 643189.f1.zip › Supplementary_Figure_2.pdf]
